# Editorial: Involvement of Tanycytes in the Neuroendocrine Control of Energy Homeostasis

**DOI:** 10.3389/fendo.2020.00464

**Published:** 2020-07-22

**Authors:** Fanny Langlet, Juan C. Sáez, María A. García-Robles

**Affiliations:** ^1^Faculty of Biology and Medicine, Center for Integrative Genomics, University of Lausanne, Lausanne, Switzerland; ^2^Departamento de Fisiología, Facultad de Ciencias Biológicas, Pontificia Universidad Católica de Chile, Santiago, Chile; ^3^Centro Interdisciplinario de Neurociencias de Valparaíso, Instituto de Neurociencias, Universidad de Valparaíso, Valparaíso, Chile; ^4^Departamento de Biología Celular, Facultad de Ciencias Biológicas, Universidad de Concepción, Concepción, Chile

**Keywords:** nutrient sensing, tanycyte glucose sensing, endocannabinoid system (eCB), leptin signaling, hypothalamo-hypophyseal portal system, tanycyte subpopulations

In the last few years, research on the central control of energy homeostasis has been unveiling the crucial role of glial cells, providing us a repository of new molecular targets to design therapeutic treatments for metabolic diseases. Among the glial cells involved in the regulation of food intake, energy expenditure, and glucose homeostasis, tanycytes are in the spotlight due to their privileged position within the brain and their wide spectrum of functions. As specialized ependymoglial cells whose cell bodies form the wall of the third ventricle, tanycytes extend their long processes within hypothalamic areas involved in metabolic control and reach close proximity with blood vessels through their end feet. Tanycytes are consequently considered an interface between the periphery, the cerebrospinal fluid (CSF), and the brain parenchyma. In this strategic position, tanycytes are able to regulate the access of metabolic information into the mediobasal hypothalamus by (1) transporting nutrients and hormones toward metabolic neuronal circuits, (2) sensing the metabolic information, and (3) appropriately releasing cytokines, nutritional metabolites, and/or gliotransmitters to modulate the activity of hypothalamic neurons. Conversely, tanycytes also control brain → periphery exchanges by regulating the secretion of key metabolic neurohormones, such as corticotropin-releasing hormone and thyrotropin-releasing hormone, in the portal vessels of the median eminence (1). Finally, tanycytes are also known for their role as neural progenitor cells within the hypothalamus, allowing regeneration and plasticity of metabolic neuronal circuits (2). Interestingly, tanycytes modulate these multiple functions depending on the physiological state of the individual in order to dynamically control energy balance by generating adaptive responses to acute nutritional challenges (3). The Research Topic “Involvement of tanycytes in the neuroendocrine control of energy homeostasis” aims to report recent advances showing the roles of tanycytes in the neuroendocrine control of energy homeostasis. This topic—containing both review articles and original research contributions—covers a variety of tanycyte functions presented above, as well as different methodologies—ranging from omics to murine physiology—currently used.

Elizondo-Vega et al. provide an overview of tanycyte nutrient sensing in the regulation of metabolic homeostasis. Tanycytes respond to nutritional signals (i.e., glucose, amino acids, fatty acids, and vitamins) by different mechanisms, including calcium signaling, metabolic shift, and/or changes in functional properties. This review highlights the need for more detailed analysis about metabolic and signaling pathways activated by nutrient sensing in tanycytes, as well as functional studies connecting tanycyte nutrient sensing with hypothalamic functions in physiological and pathological context. First, signaling pathways involved in tanycyte glucose sensing are further detailed by Salgado et al.. Hypothalamic tanycytes express several proteins involved in the glucose sensing mechanisms, in particular glucokinase and its regulator glucokinase regulatory protein (GKRP). Described in the liver, GKRP binds glucokinase and holds it inactive in the nucleus when glucose levels are low, which allows its rapid export and activation when glucose concentration rises. Hypothalamic tanycytes display an inverse dynamic in response to glucose: a rise in glucose levels drives nuclear compartmentalization of glucokinase. The authors reveal here that this differential glucokinase subcellular localization in tanycytes relies, at least in part, on the relative expression between glucokinase and GKRP and the regulation of GKRP expression and/or activity in tanycytes. Secondly, the impact of tanycyte glucose sensing on neuronal functions and physiology is reported in a functional study by Palma-Chavez et al.. The authors describe the role of the tanycyte endocannabinoid system in the modulation of arcuate neurons. The deletion of 2-arachidonoylglycerol-producing enzyme, diacylglycerol lipase-alpha (DAGLα), in tanycytes altered the usual response to CSF glucose in terms of neuropeptides produced by arcuate neurons.

In addition to nutrients, recent studies have reported that tanycytes actively transport and sense metabolic hormones, such as leptin or ghrelin. A contrasting view is presented here by Yoo et al. who report undetectable expression of leptin receptor in tanycytes as well as a tanycyte-independent regulation of some hypothalamic neuronal functions modulated by leptin signaling. Changes in cell signaling and gene expression observed in tanycytes in response to leptin are proposed to result from indirect regulations.

By projecting their endfeet in contact with fenestrated vessels in the external zone of the median eminence, tanycytes also control the release of neurohormones into the hypothalamic-hypophyseal portal system. Rodríguez-Rodríguez et al. review here the control of thyrotropin-releasing hormone release, involved in the regulation of energy expenditure through the control of basal metabolic rate and thermogenesis. Tanycytes express a repertoire of proteins involved in transport, sensing, and metabolism of thyroid hormones necessary to coordinately control the activity of the hypothalamic–pituitary–thyroid axis. Alterations in energy balance regulate the expression and activity of these proteins, making median eminence tanycytes a pivot for thyroid-related regulation of energy balance.

This adaptability to the metabolic state is a key feature for tanycytes in the regulation of energy balance. It partly relies on an accurate regulation of gene expression profiles in different tanycyte subpopulations (Langlet). Tanycytes are characterized by their own molecular signature, which is mostly associated with their diverse physiological functions, and the detection of variations in nutrient/hormone levels leads to adequate modulation of their genetic profile in order to adapt tanycyte functions and to finally regulate energy homeostasis.

Overall, significant advances have been achieved in the understanding of tanycyte functions in the regulation of energy homeostasis. This Research Topic puts new evidence on the table, points out the current controversies in this field, highlight the missing parts, and, we hope, will encourage researchers to consider tanycytes to solve the puzzle of hypothalamic control of energy balance.

## Author Contributions

FL, JS, and MG-R wrote the paper and critically revised the manuscript. All authors contributed to the article and approved the submitted version.

## Conflict of Interest

The authors declare that the research was conducted in the absence of any commercial or financial relationships that could be construed as a potential conflict of interest.
